# Psychological Aspects in Periodontal Aesthetics: Managing Gingival Recession in Body Dysmorphic Disorder

**DOI:** 10.7759/cureus.93914

**Published:** 2025-10-06

**Authors:** Deblina Saha, Amanda A Rebello, Naina Pattnaik, Dhirendra Singh, Ayushi Biswas

**Affiliations:** 1 Department of Periodontology, Kalinga Institute of Dental Sciences, Bhubaneswar, IND; 2 Department of Psychiatry, Medical College Baroda and Sir Sayajirao General (SSG) Hospital, Vadodara, IND

**Keywords:** body dysmorphic disorder, gingival recession, gingivitis artefacta, root coverage, self-harming behaviors, self-inflicted injuries

## Abstract

Patients with body dysmorphic disorder have a pathological preoccupation with a perceived physical imperfection, which is often imagined or negligible, and seek medical or cosmetic management for the same. At times, they may engage in altering their illusory imperfections to correct their appearance, which in turn can result in injuries. Besides affecting multiple organ systems, dentists often encounter oral manifestations of self-caused injuries, which pose a diagnostic dilemma as they may resemble common periodontal conditions. The success of the intervention is also significantly influenced by whether the patient continues to practice self-harming behaviors while the cosmetic treatment is ongoing. This case demonstrates a unique connection between perio-aesthetics and psychiatric factors, which is characterized by the patient's exaggerated distress over a minor self-inflicted gingival recession defect, due to a distorted perception of self. The results of this report also indicate that psychological stabilization is necessary before the initiation of any cosmetic procedure to obtain optimal therapeutic outcomes and realistic long-term expectations, emphasizing the importance of an interdisciplinary approach.

## Introduction

Patients with body dysmorphic disorder (BDD) appear to be excessively concerned by a slight or non-apparent defect, often inconsistent with any identifiable pathology or clinical pattern. They tend to harm themselves repeatedly without any realistic justification, in an attempt to fix their perceived flaws [1]. They typically display high levels of anxiety, an intense need for medical attention, and a willingness to undergo extensive diagnostic testing. The condition is further characterized by variegated symptom presentations, prolonged recoveries, and frequent changes of treating physicians. Such clinical presentations frequently prompt dental clinicians to consider an underlying psychiatric component. From the clinical perspective, the difficulty in diagnosing BDD is due to frequent changes in symptom narratives, the emergence of new complaints following the resolution of the previous ones, and the absence of standardized diagnostic measures/tools. Along with the involvement of various organ systems, in dentistry, oral manifestations of self-inflicted injuries include ulcerations, lacerations of the tongue, and/or trauma to the buccal mucosa or gingiva (gingivitis artefacta), often leading to gingival recession and bone loss [2,3]. Such injuries can be caused by biting or scratching oneself and inserting objects such as fingernails, pens, pins, or toothpicks. However, differentiating these cases from the others remains clinically challenging, mainly because artefactual injuries may mimic common periodontal issues.

This report presents a rare case of self-inflicted gingival injury causing gingival recession in a 24-year-old man presenting with abnormal psychological behavior. The case also outlines the management of gingival recessions using the tunneling method with a connective tissue graft (CTG) following psychological support and behavioral modification, which highlights a need for a collaborative effort in diagnosing and treatment planning with different specialties.

## Case presentation

A 24-year-old male patient presented to the Department of Periodontics and Oral Implantology at Kalinga Institute of Dental Sciences, Bhubaneswar, India, with a chief complaint of gums pulling away from the lower front teeth for the past year. He was also concerned about longer-looking teeth and sensitivity in the lower front tooth region. He had recently migrated to an urban city from a tribal village to pursue his PhD and was experiencing considerable academic stress, challenges associated with cultural adaptation, and dietary changes. He reported a history of multiple dental consultations to various clinics in the city and received only symptomatic interventions without any definitive treatment. On general physical examination, the patient presented with a lean physique and appeared to be of poor build with a dull facial expression. He appeared to be visibly distressed and overly anxious regarding his oral health concerns. Extraorally, multiple linear and patchy scars were observed on the face, neck, and hands, which the patient attributed to repeated attempts to "clean" or "fix" perceived skin imperfections.

**Figure 1:**
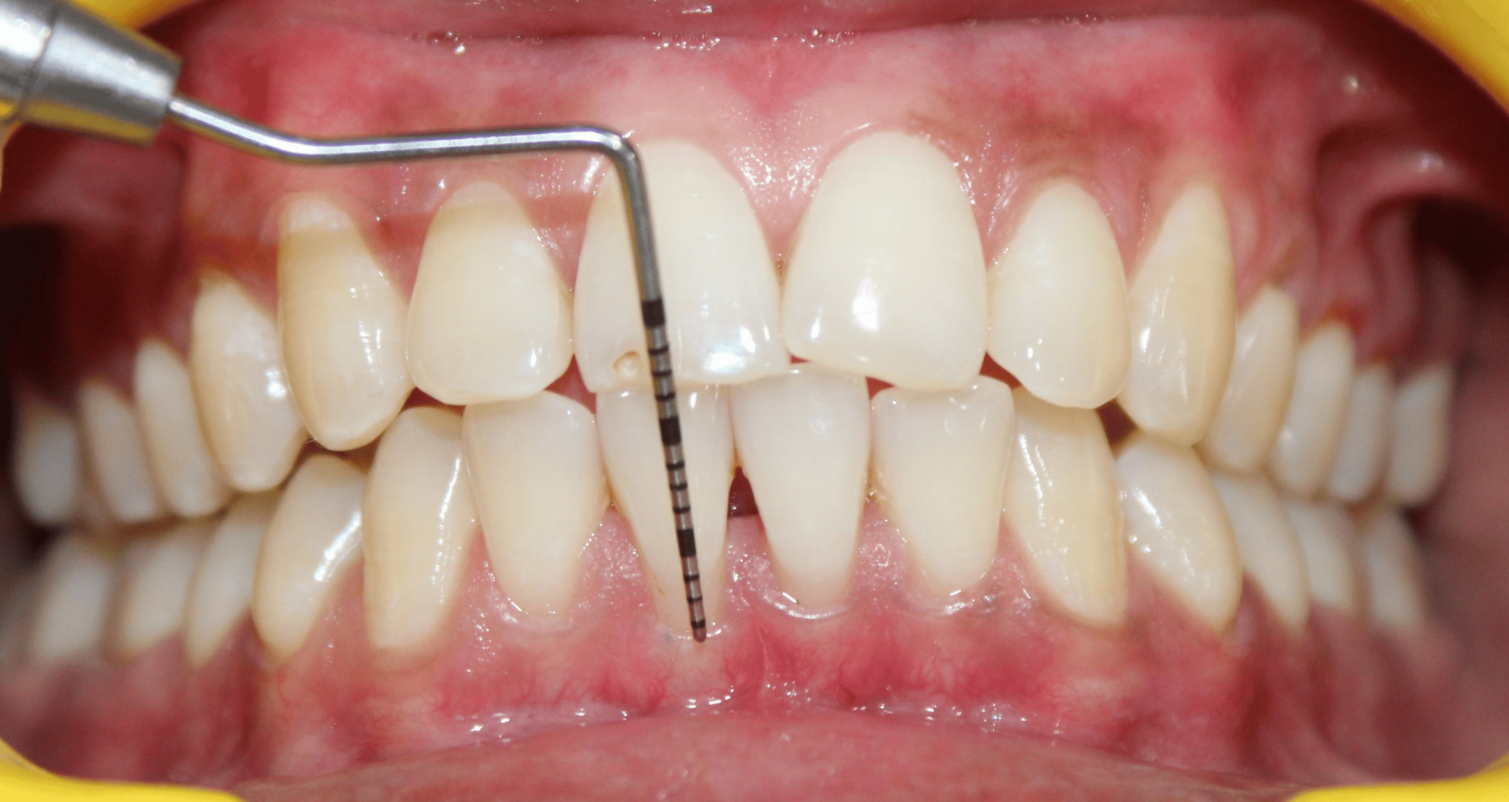
Miller class I gingival recession with respect to tooth #41 Intraoral clinical photograph showing a 3 mm gingival recession with respect to the mandibular right central incisor (tooth #41) and an interdental papillary height loss in between two mandibular central incisors (teeth #31 and #41), creating a black triangle. The recession depth is measured using a UNC-15 periodontal probe. UNC: University of North Carolina

**Figure 2:**
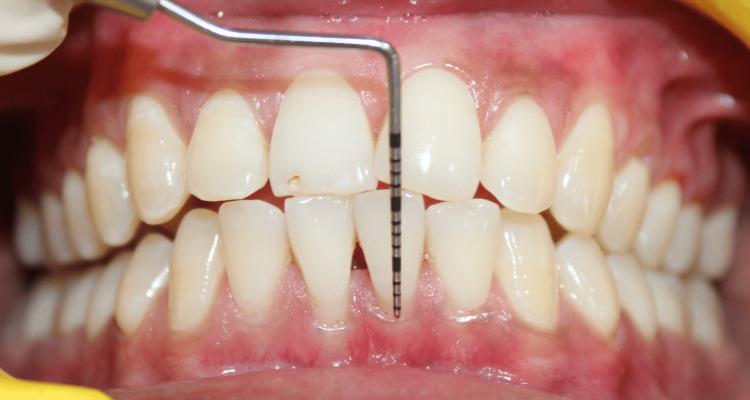
Miller class I gingival recession with respect to tooth #31 Intraoral clinical photograph showing a 2 mm gingival recession with respect to the mandibular left central incisor (tooth #31) and an interdental papillary height loss in between two mandibular central incisors (teeth #31 and #41), creating a black triangle. The recession depth is measured using a UNC-15 periodontal probe. UNC: University of North Carolina

**Figure 3:**
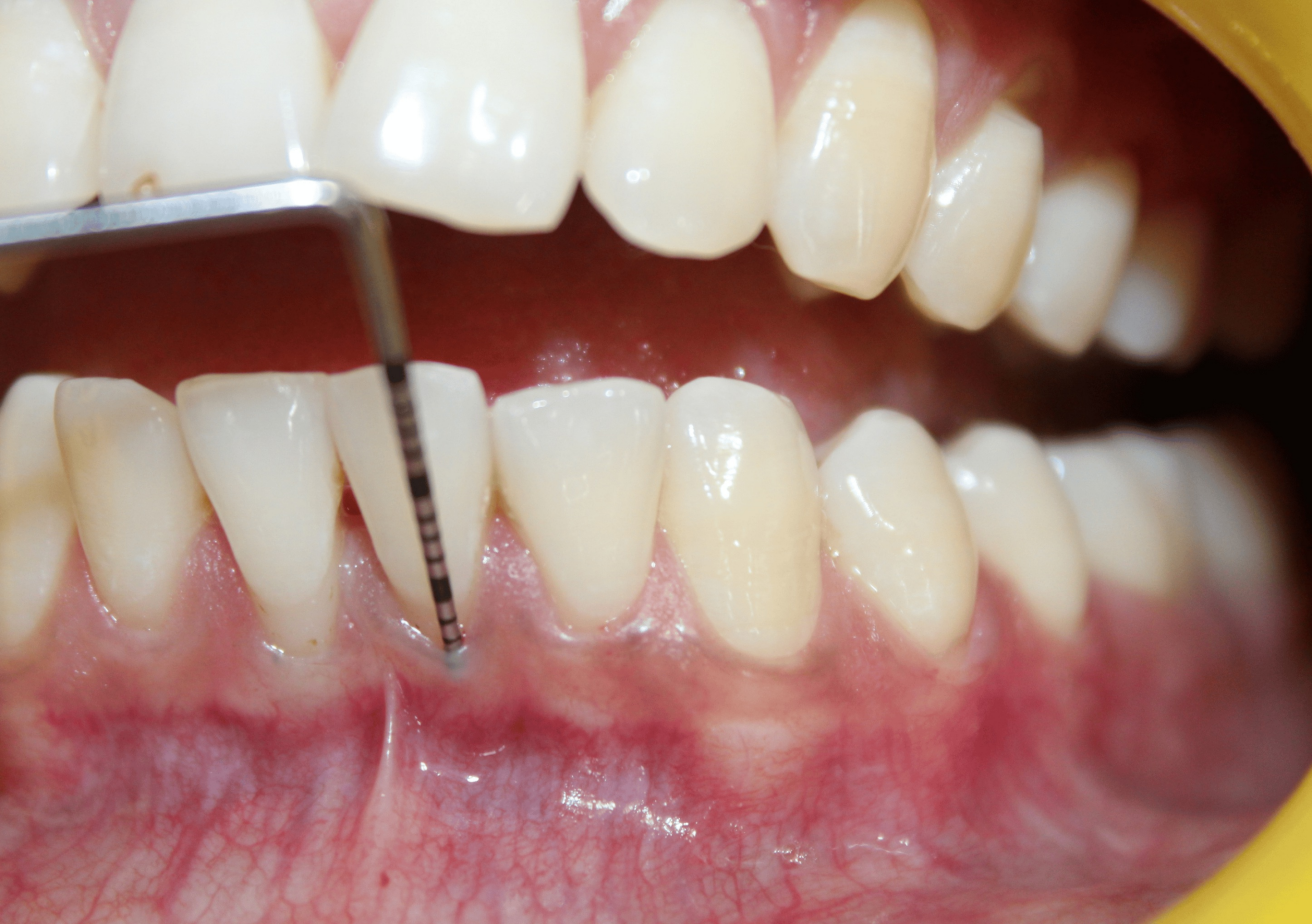
Assessment of phenotype Intraoral clinical photograph showing the thin gingival phenotype assessed by the probe transparency method. The tip of the UNC-15 periodontal probe is visible through the gingival margin, confirming the thin and delicate gingival tissue. UNC: University of North Carolina

On clinical examination, there were Miller class I gingival recession in relation to teeth #31 and #41 and a loss of interdental papillary height between the lower central incisors (Figures 1-2). Plaque deposits were minimal, and the gingival tissues exhibited characteristics of a thin tissue phenotype (Figure 3). On detailed questioning, the patient's responses reflected an extremely cautious, apprehensive attitude concerning his oral health. Despite adequate oral hygiene, the patient reported brushing 3-4 times daily with excessive force. He disclosed a history of habitual picking of the gingiva with sharp objects such as a pen, safety pin, or toothpick. This unusual behavior raised suspicion of an inexplicable medical presentation with a psychological component. Considering the exaggerated concern for oral health, repeated consultations across multiple clinics, and the self-imposed nature of gingival injury, a provisional diagnosis of gingivitis artefacta major was established, and a psychiatric referral was made.

Based on a detailed assessment, the patient was diagnosed with BDD with self-induced oral and facial injury. Psychiatric behavioral therapy was initiated with regular psychological consultations to evaluate his progress, simultaneously with periodontal treatment, including scaling and root planing, followed by a root coverage procedure. Nutritional counselling was also initiated, with attention focused on a stable cultural transition with subsequent dietary pattern modifications.

Surgical procedure

**Figure 4:**
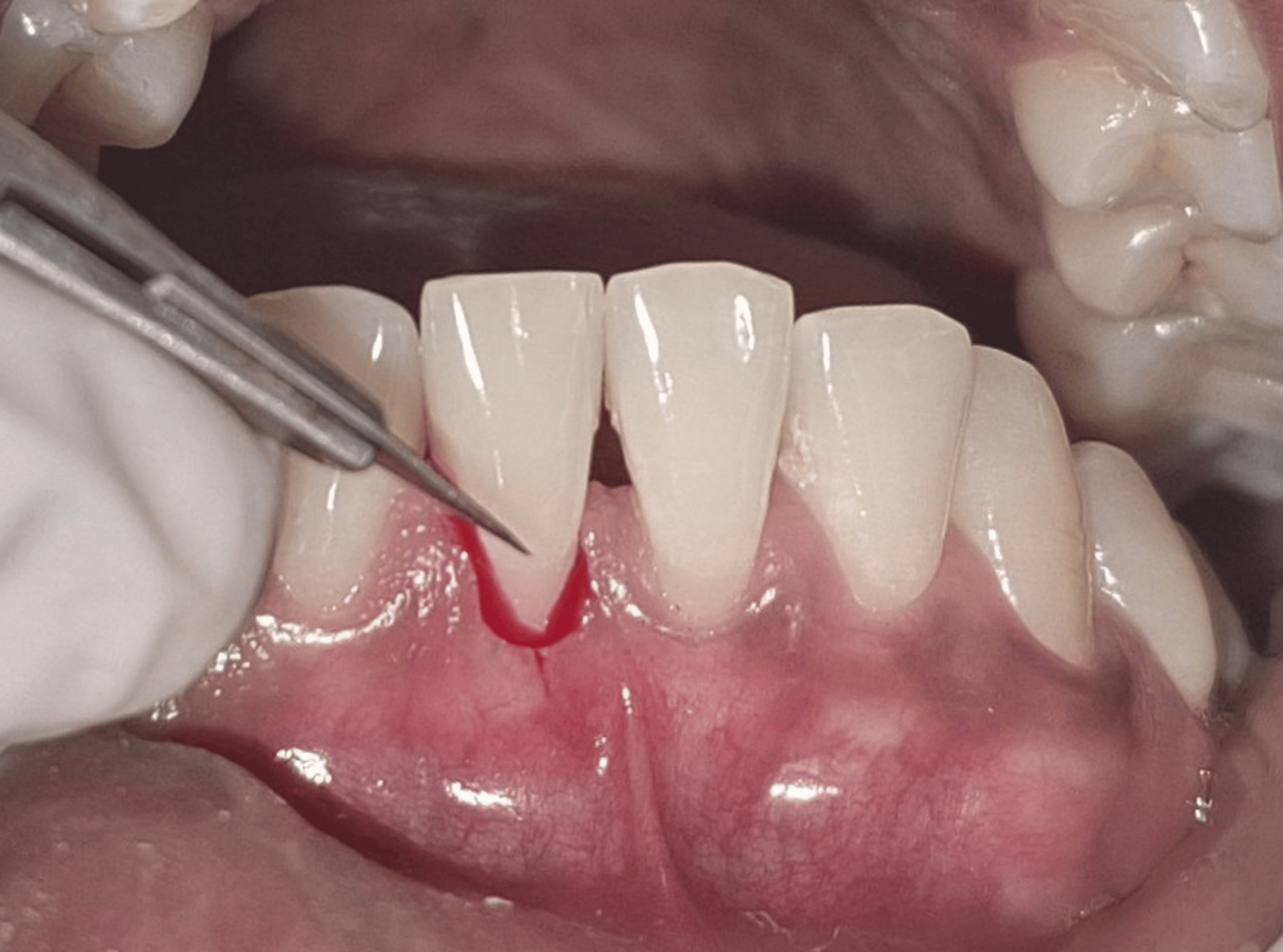
Crevicular incision placed around the gingival margin Intraoral clinical photograph showing the placement of a crevicular incision using a blade breaker and a #11 blade. The instrument is engaged along the gingival margin to initiate the incision.

Following the administration of local anaesthesia, a crevicular and vertical vestibular incision was made, approximately 8-10 mm apical to the mucogingival junction (Figure 4).

**Figure 5:**
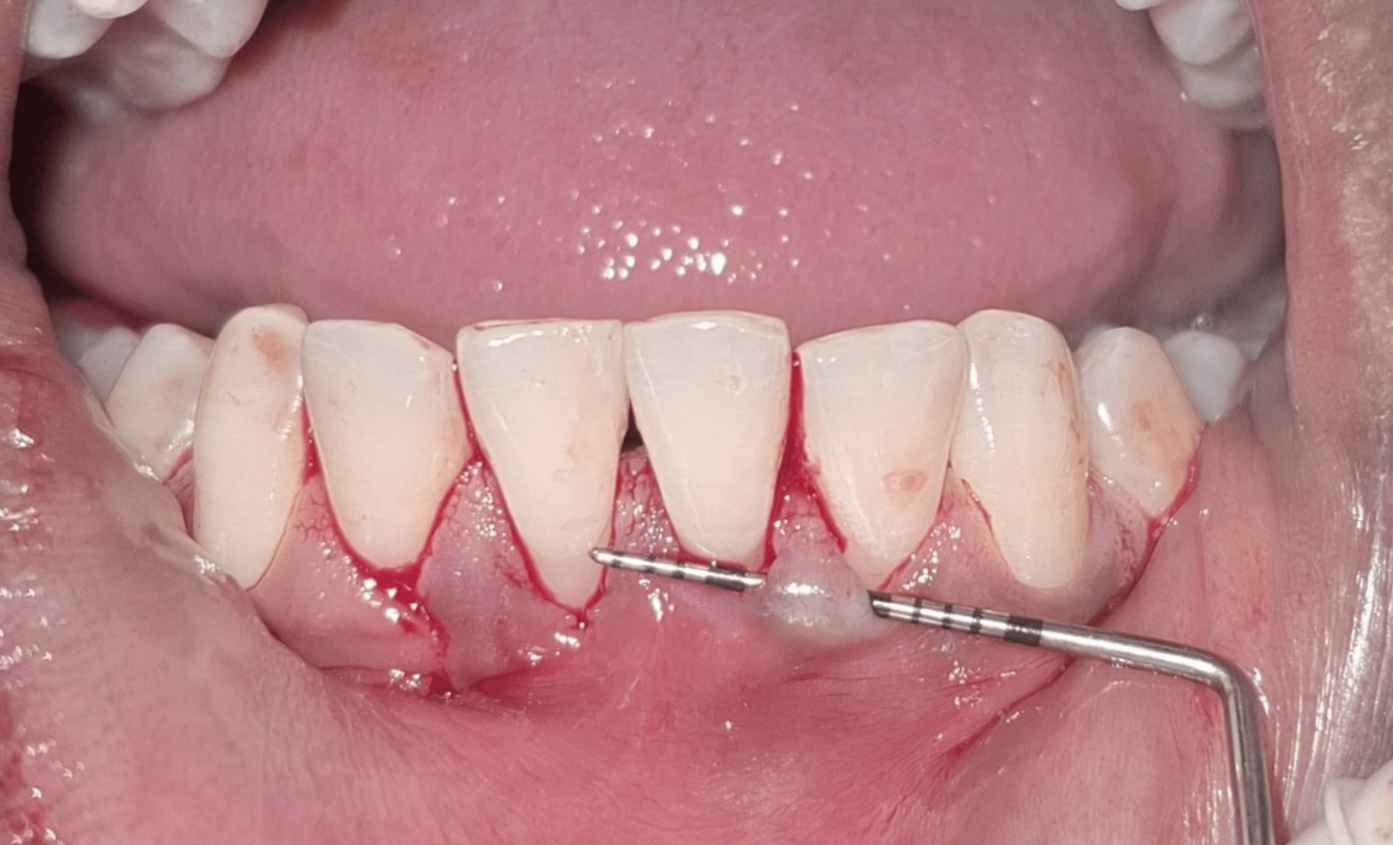
Demonstration of tunneling Intraoral clinical photograph showing the tunneling performed using a tunneling instrument following a vestibular incision placed approximately 8-10 mm apical to the mucogingival junction in the region between teeth #41 and #42. A subperiosteal tunnel is demonstrated with the aid of a UNC-15 periodontal probe at the region between #31 and #32. UNC: University of North Carolina

A subperiosteal tunnel was created using specially designed tunneling instruments, carefully extending coronally to involve the interdental papillae adjacent to #31 and #41 (Figure 5). Care was taken to preserve the papillary integrity and maintain flap continuity throughout the procedure.

**Figure 6:**
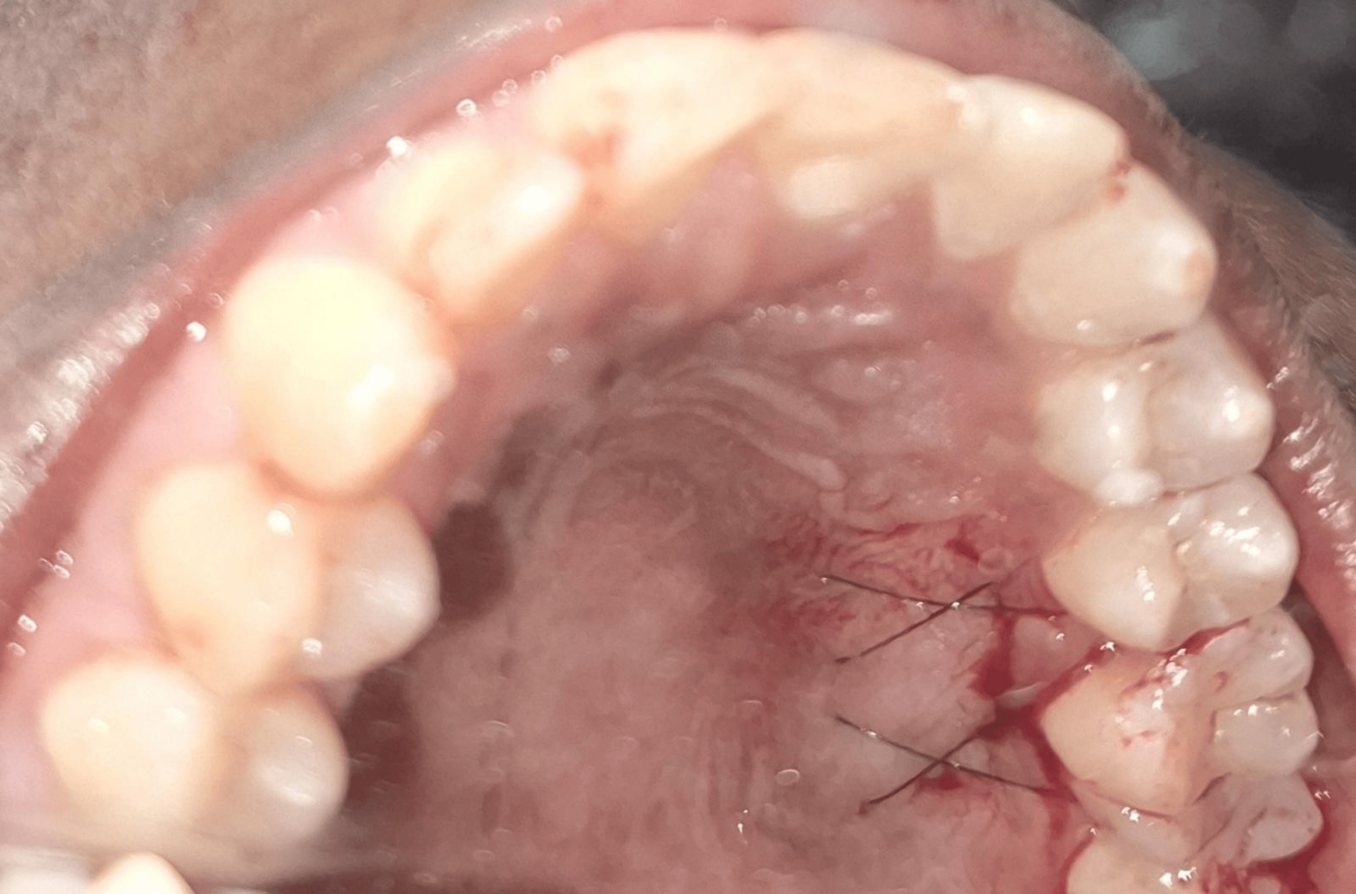
CTG-harvested donor site Intraoral clinical photograph showing the palatal donor site after harvesting a CTG using the single-incision technique. Criss-cross sutures are placed in the maxillary right second premolar and first molar region (teeth #15 and #16) for wound closure. CTG: connective tissue graft

**Figure 7:**
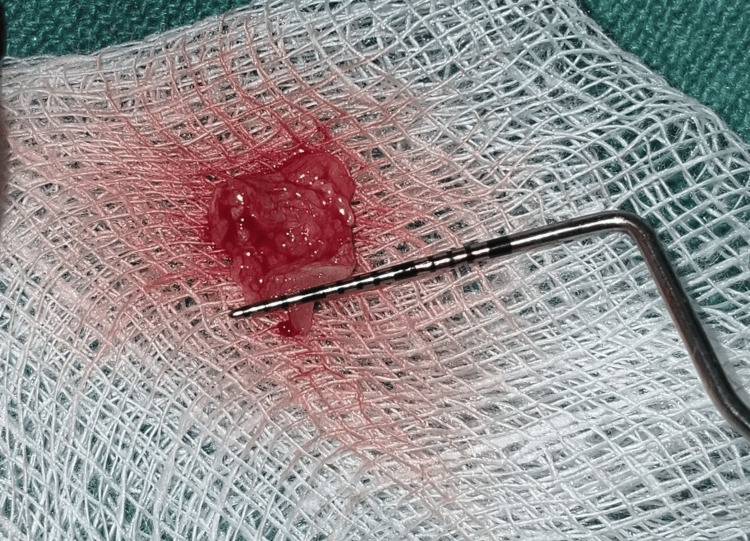
Harvested CTG measuring 7 mm in width Harvested CTG measuring approximately 7 mm in width, as assessed using a UNC-15 periodontal probe. CTG: connective tissue graft; UNC: University of North Carolina

Simultaneously, a CTG was harvested from the palatal mucosa in the premolar-molar region using the single-incision technique (Figures 6-7). A horizontal incision, parallel to the gingival margin, was made in the palatal mucosa, and a partial-thickness flap was raised. Using blunt dissection, the CTG was carefully separated from the underlying tissue and retrieved through the same incision. The donor site was sutured with 6-0 Ethilon, and an absorbable gelatin sponge was placed for haemostasis.

**Figure 8:**
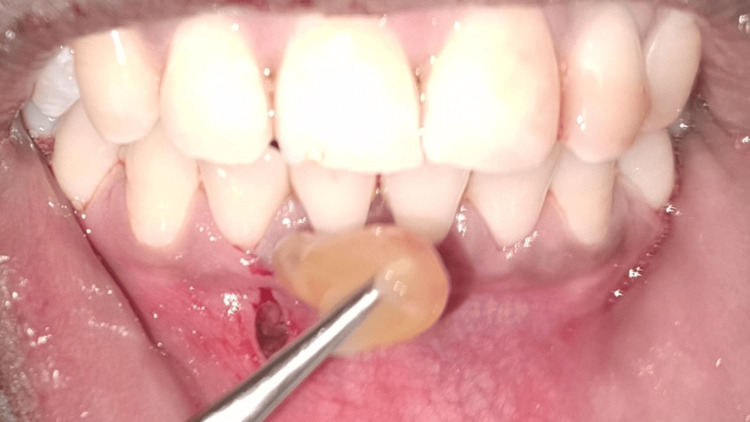
PRF application Intraoral clinical photograph showing the placement of a PRF plug into the recipient site through a vestibular incision. The PRF is adapted within a subperiosteal tunnel. PRF: platelet-rich fibrin

**Figure 9:**
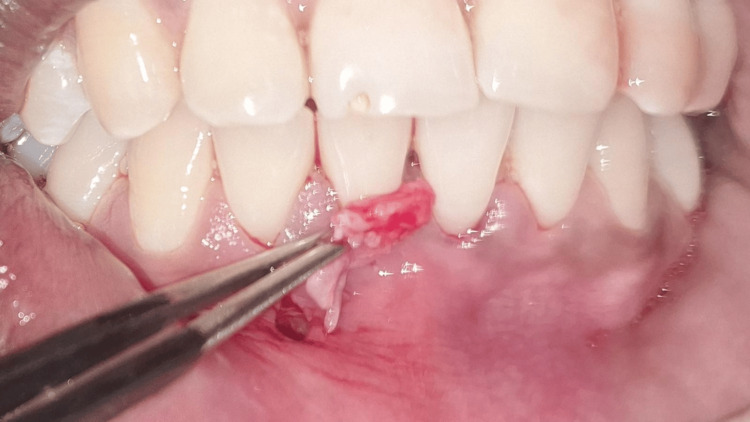
Graft insertion done Intraoral clinical photograph showing the graft insertion into the recipient site through the vestibular incision, followed by graft stabilization.

**Figure 10:**
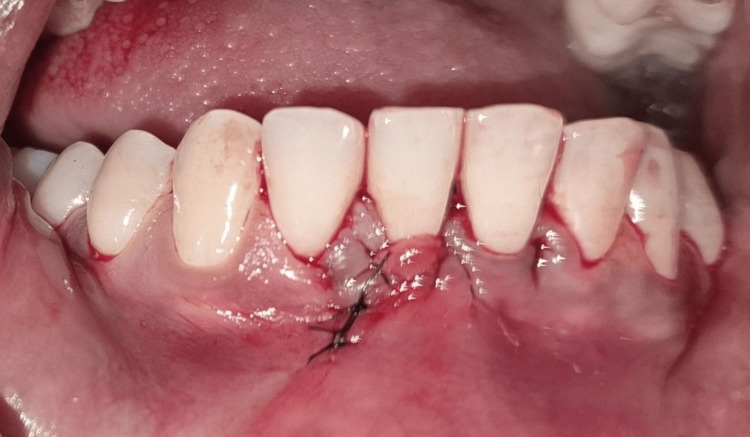
Flap advanced coronally and stabilized using sling sutures Intraoral clinical photograph showing the advancement of the flap in the coronal direction and stabilization using sling sutures using 5-0 Ethilon. Vestibular incision is sutured with interrupted sutures.

The harvested CTG was then trimmed and inserted through the vestibular access incision into the prepared tunnel following platelet-rich fibrin (PRF) plug application (Figures 8-9). The graft was positioned over the denuded root surfaces, and the flap, along with the graft, was coronally advanced. It was secured in position using sling sutures and interrupted sutures to ensure a passive, tension-free adaptation (Figure 10). The vestibular incision was also sutured, and a periodontal dressing was placed.

Postoperative instructions were provided, including restricted brushing and manipulation of the surgical area, chlorhexidine mouthwash rinsing, and analgesics to be taken as required.

**Figure 11:**
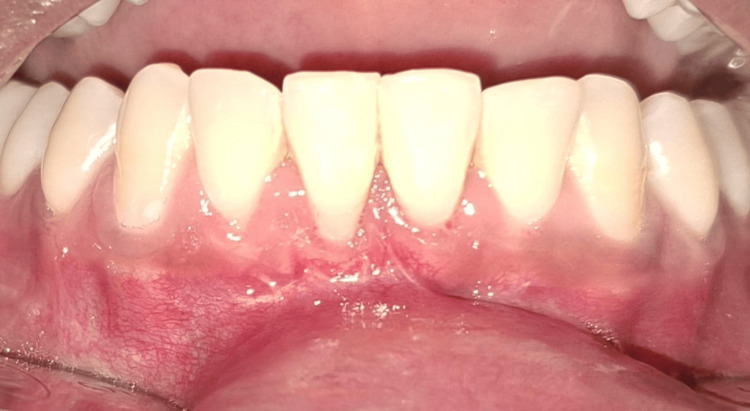
Post-op six months Clinical photograph at six months postoperatively, demonstrating noticeable improvement in key clinical parameters, including increased gingival thickness, enhanced interdental papillary height, and reduction in gingival recession compared to baseline (Figure 1).

**Figure 12:**
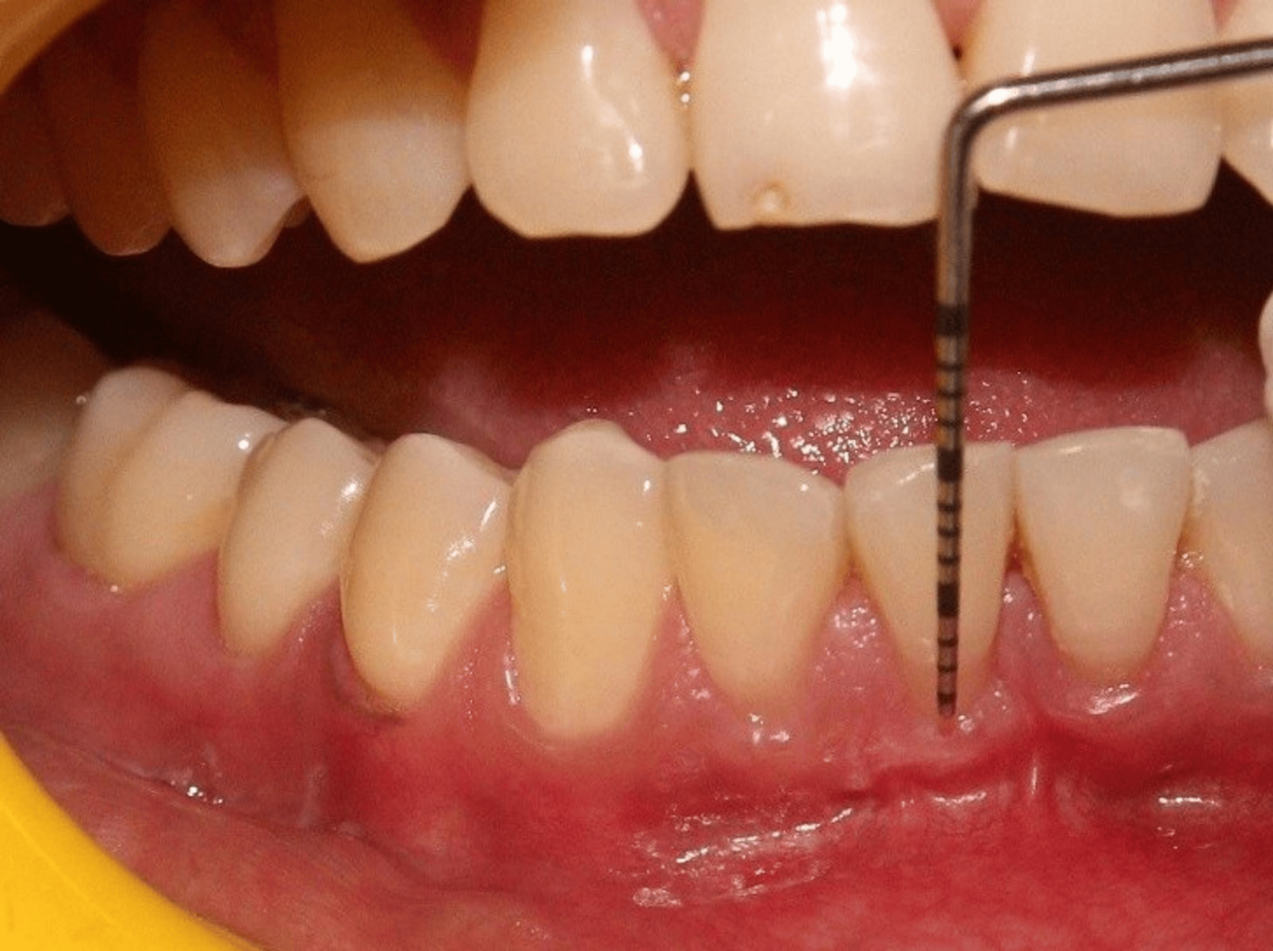
Post-op one year Clinical photograph at one year postoperatively, illustrating the sustained improvement in clinical parameters, including stable gingival phenotype, near-complete restoration of interdental papillary height, and a marked reduction in gingival recession, with a residual recession depth of 1.5 mm with respect to tooth #41, measured using a UNC-15 periodontal probe, compared to baseline (Figure 1) and six-month follow-up (Figure 11). UNC: University of North Carolina

**Figure 13:**
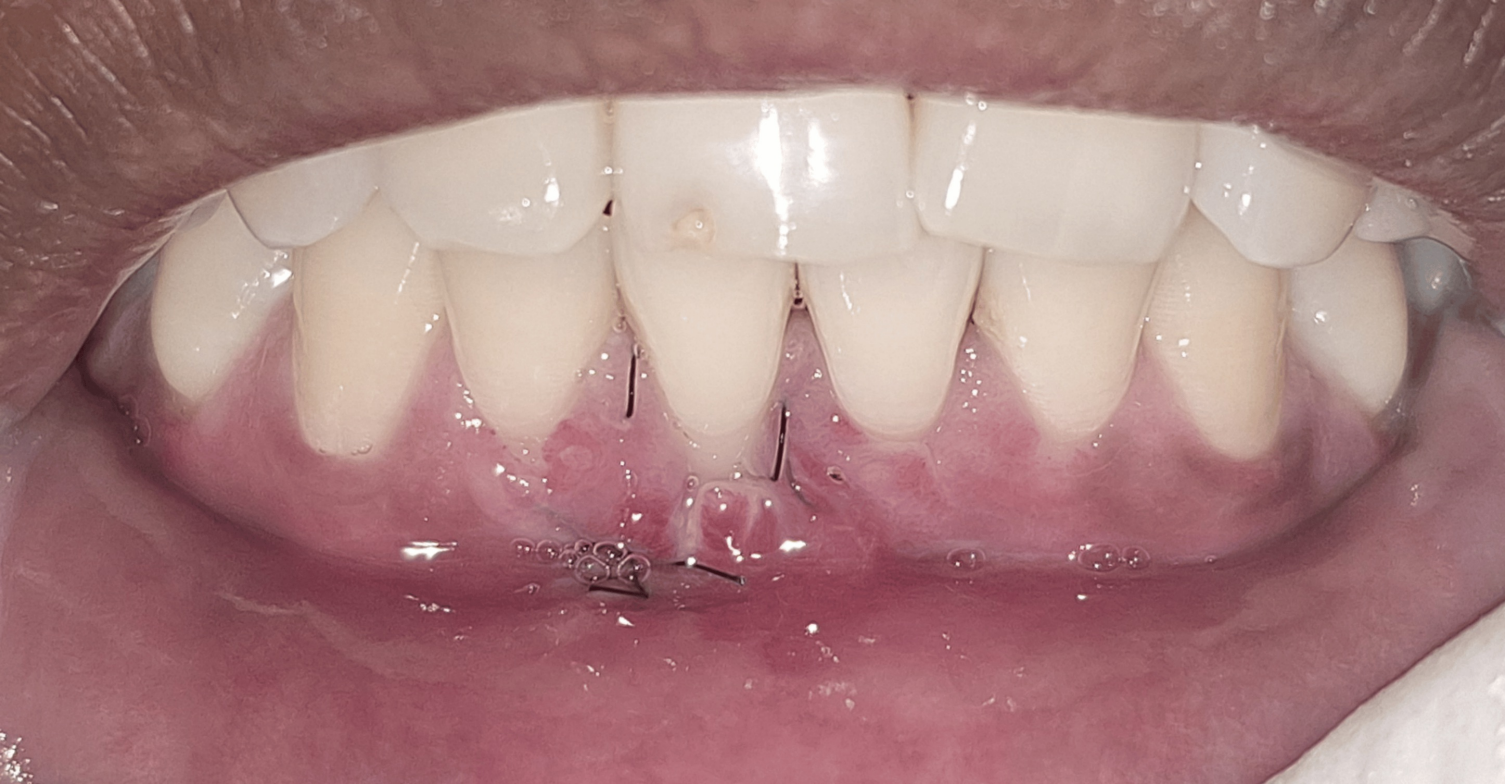
Early suture loosening on the 10th postoperative day Clinical photograph on the 10th postoperative day showing early suture loosening at the surgical site, attributed to self-inflicted manipulation, resulting in partial flap displacement.

A detailed comparative analysis of clinical outcomes was performed at baseline, six months postoperatively, and one year postoperatively. This analysis focused on key parameters, including the gingival phenotype, the height of the interdental papilla, and the extent of gingival recession. Over the follow-up period, notable improvements were observed, such as the following: the gingival phenotype demonstrated increased thickness and resilience, the interdental papillary height showed significant recovery, and the recession depth was reduced consistently across treated sites. Representative clinical photographs documenting these changes are provided from baseline (Figure 1), through six months post-op (Figure 11), to one year post-op (Figure 12), illustrating the progressive clinical improvements achieved with the surgical intervention. Furthermore, suture loosening was noted on the 10th postoperative day at the surgical site (Figure 13), leading to premature flap displacement, which may have contributed to the partial root coverage observed with respect to tooth #41.

## Discussion

Gingival recession is a common clinical finding in periodontal practice, often associated with aesthetic concerns, root sensitivity, and plaque retention. While surgical root coverage procedures are standard in managing localized recession defects, their management becomes significantly more complex when intertwined with underlying psychological disorders. A summary of previously reported cases of gingival recession caused by self-inflicted injury is presented in Table 1.

**Table 1:** Summary of previously reported cases of self-inflicted gingival recession This table outlines the clinical presentations, causes, and findings from earlier case reports and series, including self-inflicted gingival injuries due to nail scratching or factitious habits as described by Pattnaik et al. [3], Maalem et al. [4], Ansari et al. [5], and Dilsiz and Aydin [6].

S. no.	Author/year	Cause of gingival recession	Findings
1	Pattnaik et al./2015 [3]	Self-inflicted gingival injury	This case series describes three young male patients with self-caused gingival injuries (gingivitis artefacta major) linked to underlying emotional disturbances. Initial periodontal care, followed by psychiatric consultation and cognitive behavioral therapy, helped control their self-injurious habits before definitive dental treatment
2	Maalem et al./2024 [4]	Habitual fingernail scratching	This case highlights a rare instance of self-inflicted gingival recession affecting primary maxillary incisors in a five-year-old child, managed successfully through oral hygiene care, behavioral guidance, and long-term follow-up (over 16 months)
3	Ansari et al./2024 [5]	Factitious nail scratching habit	This paper reports the diagnosis and management of a 16-year-old male patient with bilateral mandibular canine gingival recession caused by habitual nail scratching. A combined behavioral, psychological, and surgical approach using a modified lateral pedicle flap with/without an amnion-chorion membrane achieved stable soft tissue health at two years
4	Dilsiz and Aydin/2009 [6]	Habitual fingernail scratching	This case report describes a 14-year-old girl with gingival recession in the mandibular anterior region caused by habitual fingernail trauma. A combined approach of oral hygiene instruction, debridement, psychological support, and periodontal surgery using free gingival graft achieved complete root coverage and stable periodontal health

This case illustrates a rare condition that acknowledges the intersection between periodontal aesthetics and psychiatric illness, where the patient's exaggerated concern about the minor gingival defects resulting from self-harm arose due to a distorted perception of his own appearance.

BDD, classified under obsessive-compulsive and related disorders in the DSM-5, is characterized by a persistent and intense apprehension about imagined defects or minor flaws in physical features, often not evident to others [7]. The prevalence of BDD ranges from 0.7% to 2.5% in the general population but can increase to as high as 19.2% among individuals seeking cosmetic treatments [8]. Although most documented BDD cases involve facial or dermatologic concerns, dental and periodontal aspects, particularly involving the smile zone or anterior gingiva, can serve as an obsessive fixation point. Notably, approximately 86% of BDD patients report concerns involving the teeth or facial features [8]. The central incisors (#31 and #41), being in the cosmetic zone, often attract heightened attention from BDD individuals, who may engage in excessive oral hygiene practices, such as forceful brushing or traumatic manipulation, further exacerbating gingival recession. Diagnosing BDD remains clinically challenging due to the lack of a universally accepted diagnostic tool. However, screening questionnaires, detailed medical and psychiatric anamnesis, and an awareness of unrealistic aesthetic expectations or multiple prior consultations can aid in early recognition [9-11].

BDD is frequently considered a relative contraindication for elective aesthetic treatments, given the high likelihood of dissatisfaction and requests for repeated interventions [12,13]. However, there is insufficient valid evidence to support the hypothesis that patients with psychiatric disorders have higher rates of dissatisfaction following surgical treatment [14]. In moderate to severe cases, referral for psychiatric evaluation and management using cognitive behavioral therapy (CBT) and/or selective serotonin reuptake inhibitors (SSRIs) is recommended prior to any surgical procedure [15].

Given the patient's psychological vulnerability, the tunneling technique was selected for its minimally invasive approach and superior aesthetic outcomes. The use of CTG provided additional soft tissue thickness and enhanced stability, which are crucial in high-risk cases with self-inflicted trauma. Surgical intervention was delayed until some level of psychiatric stabilization was achieved, and treatment planning was done collaboratively with the mental health team to ascertain that the patient's expectations were practical and that he was capable of adhering to proper postoperative care. Progressive alterations in recorded clinical parameters from baseline through six-month and one-year postoperative evaluations are summarized in Table 2.

**Table 2:** Comparative analysis of clinical outcomes at baseline, post-op six months, and post-op one year Comparative analysis of clinical outcomes at baseline, six months postoperatively, and one year postoperatively, demonstrating changes in gingival phenotype, improvement in interdental papillary height, and reduction in recession depth. Corresponding clinical photographs are presented in Figure 1 (baseline), Figure 11 (six months), and Figure 12 (one year).

Clinical outcome	Pre-op (Figure 1)	Post-op six months (Figure 11)	Post-op one year (Figure 12)
Change in gingival phenotype	Thin phenotype with tissue translucency and scalloped gingival margin	Increased gingival thickness and width of keratinized tissue	Stable, thick phenotype with well-contoured margins
Regeneration of lost interdental papilla	Open interdental space with black triangle formation due to loss of papillary height and volume	Complete fill of interdental space with increased papillary height	Slight loss of regenerated papillary height, likely associated with recurrent calculus deposits or self-inflicted injury
Reduction in gingival recession depth	3 mm of recession depth with respect to #41 and 2 mm of recession depth with respect to #31	2 mm of recession depth with respect to #41 and complete root coverage achieved with respect to #31	1.5 mm of recession depth with respect to #41 and complete root coverage achieved with respect to #31

At the one-year follow-up, the clinical outcome was notably positive. Complete root coverage was achieved for tooth #31, while partial root coverage was noted for tooth #41, along with a significant gain in interdental papillary height and favorable phenotype modification, which appeared thicker and more resilient (Figure 12). Importantly, from a psychiatric perspective, the patient demonstrated a marked reduction in an intrusive preoccupation with gingival visual characteristics and discontinuation of self-injurious oral habits, which was assessed using standardized clinical examinations and questionnaires to evaluate the psychiatric progress. This improvement was attributed to ongoing psychiatric therapy, including cognitive behavioral support, which ran parallel with the periodontal treatment. However, due to limitations in clinical assessment scores and patient confidentiality considerations, this diagnosis could not be definitively included within the scope of this current report.

However, a slight loss of interdental papillary volume was noted at the follow-up. It could be attributed to individual healing variability or early mechanical stress. Additionally, loosening of sutures was observed on the 10th postoperative day at the operated site, resulting in early flap displacement, which raised the possibility of self-inflicted manipulation, especially given the patient's psychiatric background (Figure 13). In this context, the clinician should remain alert as patients with BDD may exhibit obsessive behaviors, such as repeated inspection or interference with healing sites, which is not uncommon and must be carefully monitored.

This case highlights the need for coordinated care in the management of gingival recession in patients with BDD. Dentists should remain vigilant when patients show disproportionate concern over minor aesthetic issues, a history of multiple consultations, or persistent dissatisfaction despite adequate clinical outcomes. A collaborative referral to mental health professionals is not only warranted but essential in accurately diagnosing the underlying aetiology, predicting the treatment outcome, preventing over-treatment, and supporting the patient's overall well-being.

As this report describes a single case, the generalizability of its findings is limited. Larger studies with greater sample sizes and longer follow-up periods are required to substantiate these results. In addition, standardized indices such as plaque index, bleeding on probing, and keratinized tissue width were not recorded, which represents a limitation of this report.

Ultimately, the management of such patients requires the integration of clinical expertise, psychological assessment, and therapeutic communication strategies, emphasizing that effective treatment must encompass both tissue regeneration and psychosocial well-being.

## Conclusions

This case highlights the complex interplay between psychological factors and periodontal health, demonstrating that successful management of self-inflicted gingival injury requires an integrated approach in which psychiatric evaluation and precise periodontal therapy are essential. 

Early identification of self-inflicted behaviors, careful monitoring of postoperative healing, and tailoring periodontal interventions to individual psychological and behavioral factors were crucial in optimizing results. Clinicians should remain vigilant for signs of patient-induced trauma and place emphasis on clear postoperative instructions along with appropriately scheduled follow-up visits to ensure long-term stability. Patients should be addressed holistically, rather than focusing solely on the localized clinical defect. Therefore, addressing both the mental health component and the clinical defect was critical to achieve stable and satisfactory outcomes, underscoring the importance of an interdisciplinary collaboration in similar cases.

However, as this report represents a single case study, its findings carry inherent limitations, and larger studies with long-term follow-up are required to validate these observations. From such evidence, a standardized screening checklist could be developed, integrating both psychiatric and periodontal components, to facilitate the early detection and comprehensive management of similar conditions.
